# Grape-Seed-Derived Procyanidin Attenuates Chemotherapy-Induced Cognitive Impairment by Suppressing MMP-9 Activity and Related Blood–Brain-Barrier Damage

**DOI:** 10.3390/brainsci12050571

**Published:** 2022-04-28

**Authors:** Chao Song, Chao Gao, Zhenxin Wang

**Affiliations:** 1 Department of Oncology, First Affiliated Hospital of Soochow University, Suzhou 215006, China; 20184132216@stu.suda.edu.cn; 2 Department of Oncology, Shanghai East Hospital, Tongji University School of Medicine, Shanghai 200120, China; 3 Department of Oncology, The Affiliated Hospital of Xuzhou Medical University, Xuzhou 221002, China; 770020210081@xzhmu.edu.cn

**Keywords:** cisplatin, chemotherapy, MMP-9, RAW264.7 cells, procyanidin, blood–brain barrier (BBB), chemotherapy-induced cognitive impairment (CICI)

## Abstract

(1) Background: Chemotherapy-induced cognitive impairment (CICI) is often observed in cancer patients and impairs their life quality. Grape-seed-orientated procyanidin has been shown to have anti-inflammatory and neuroprotective effects, yet its effects in preventing CICI have not been investigated. (2) Method: Adult male mice received 2.3 mg/kg cisplatin or saline injections for three cycles consisting of five daily injections followed by 5 days of rest. Procyanidin or saline was administered 1 h prior to cisplatin treatment. Cognitive testing, gelatin zymography, and blood–brain-barrier (BBB) penetration tests were performed after treatment cessation. RAW264.7 cells were treated by stimulated supernatant of SHSY5Y cells. In addition, high-mobility group protein B1 (HMGB1) expression and MMP-9 activity were tested. (3) Results: Repeated cisplatin treatment increased BBB penetration, MMP-9 activity, impaired performance in contextual fear conditioning, and novel object recognition tasks. The knockout of MMP-9 rescues cognitive impairment and cisplatin-induced upregulation of HMGB1 in SHSY5Y cells. HMGB1/TLR4/IP3K/AKT signaling contributes to the increased MMP-9 activity in RAW264.7 cells. Procyanidin treatment attenuates MMP-9 activity, BBB damage, and CICI. (4) Conclusions: The results indicated that MMP-9 activation and BBB disruption is involved in CICI. Procyanidin may effectively alleviate the harmful effects of cisplatin.

## 1. Introduction

Chemotherapy drugs are some of the most effective forms of anticancer treatment. With the advent of chemotherapeutic agents, cancer patients have had a huge increase in survival rates. However, they are related to many adverse side effects, such as cardiotoxicity, peripheral neurotoxicity, and hepatotoxicity. Recently, chemotherapy-induced cognitive impairment (CICI), also known as “chemobrain”, is becoming increasingly recognized [1,2]. Moreover, it is often observed in cancer patients during and even postchemotherapy [3]. The impaired cognitive domains of CICI include verbal and visual memory, psychomotor function, difficulty in learning, and poor attention span. In clinical settings, CICI is a common neurological complication after chemotherapy, could affect up to 78% of treated patients, is closely related to impaired life quality, and hampers the achievements of occupational goals [4]. Therefore, it is urgent to understand the underlying mechanisms and find a safe and effective strategy to prevent cognitive impairment.

There are several potential mechanisms and etiologies contributing to CICI, including direct neurotoxicity, increased oxidative stress, disruption of the blood–brain barrier (BBB), and secondary neuroinflammatory response. For instance, cisplatin can exert direct neurotoxic injury. It is a DNA targeting agent, forming toxic platinum DNA adducts and inducing necrosis and apoptosis [5]. The damaged cells lead to the upregulation of the peripheral circulating cytokines, which recruit immune cells to induce neuronal apoptosis, causing cognitive impairment even at low concentrations [6,7,8]. BBB disruption, leading to peripheral chemotherapy drugs and pathogens precisely entering neural tissue, is believed to be pivotal in the pathology and progression of many cognitive-decline-related diseases [9,10]. Therefore, understanding the mechanisms of chemotherapeutic-drug-induced changes in BBB integrity will provide novel insights into the prevention of CICI.

It is widely known that matrix metalloproteinase-9/2 (MMP-9/2) is closely related to the breakdown of BBB and thereby facilitates neuronal and synaptic dysfunction in many other diseases. MMP-9/2 could be produced by many cells, including macrophages. It not only cleaves vascular basal lamina and/or tight junctions between cells and then enhances BBB permeability but also is known as a major and apparently unique player in brain physiology and pathology [11,12]. MMP-9 promotes inflammatory cell infiltration, and its role in neuroinflammation and cognitive decline has been well-reviewed [13]. Interestingly, the upregulation of MMP-9 has recently been reported to relate to chemotherapy-induced peripheral neurotoxicity (CIPN) [14,15], while the role of MMP-9/2 in CICI has not yet been evaluated.

It is of note that cisplatin induces cell death and promotes damage-associated molecular patterns (DAMPs) released in peripheral blood systems. DAMPs are the most crucial inflammatory and immune response triggers [15,16]. For instance, the high-mobility group protein B1 (HMGB1) and Toll-like receptor (TLR2/TLR4) signaling cascade contributes to phenotypic switching to M1 macrophage polarization and upregulation of MMP-9 (refs. [17,18]), which is related to cognitive impairment disease. The receptor for advanced glycation end-product (RAGE) is another candidate for upregulating MMP-9 signaling. It is the signal transduction receptor that senses the signaling molecules including HMGB1 and oxidative stress and could promote nuclear factor-kB (NF-kB) activation and secretion of various cytokines and MMP-9. However, the MMP-9 in macrophages which contributes to the development of CICI has not yet been fully understood.

Several natural products with neuroprotective properties were confirmed in the amelioration of cisplatin neurotoxic action [7]. For example, the administration of chemotherapeutic drugs, N-acetylcysteine [19], ginsenoside Rg1 [20], or curcumin, has been reported to attenuate cisplatin-induced cognitive decline [21]. Procyanidin is a flavonoid mainly found in grape seeds and green tea. It has been demonstrated to have a powerful oxygen radical absorbance capacity and could attenuate neuropathic pain and chemotherapy-induced peripheral neuropathy (CIPN) [14,22]. In addition, previous studies have reported that procyanidin has a potent inhibitory effect on MMP-9 and NF-κB signaling in macrophages to alleviate neuropathic pain or inflammatory bowel disease [23]. It also has been reported to inhibit the production of the HMGB1 danger signal by activated monocytes [24].

To date, there has been no study focusing on whether grape seed extracts could mitigate CICI. The contribution of MMP-9-related BBB integrity changes in CICI has not yet been investigated. In the present study, we focus on the changes of MMP-9/2 activity and BBB integrity induced by cisplatin. In addition, we further test the role of HMGB1 signaling in macrophage MMP-9/2 upregulation. Finally, we will evaluate the role of procyanidin in attenuating CICI and the generation of MMP-9 and BBB permeation.

## 2. Materials and Methods

### 2.1. Animals

All animals were handled according to good animal practice. Animal work was approved by the appropriate committee, Xuzhou Medical University, Xuzhou, China. Wild-type mice (C57BL/6J, 12–14 wk old) were provided by the Experimental Animal Center at Xuzhou Medical University, Xuzhou, China. MMP-9 mutant (MMP-9^−/−^) mice were purchased from the Jackson Laboratory (Bar Harbor, ME, USA). The mice were housed five in a cage under pathogen-free conditions during a 12 h light/dark cycle and under controlled temperature (22 ± 2 °C).

### 2.2. Drug and Chemicals

Procyanidin was purchased from Aladdin Co. Ltd. (Shanghai, China). Secondary antibodies were purchased from Cell Signaling Technology (Beverly, MA, USA). Toll-like receptor 4 (TLR-4) inhibitor TAK-242, phosphatidylinositol 3-kinase (PI3K) inhibitor AS-605240, and protein kinase B (Akt) inhibitor GSK690693 were purchased from Selleck Chemicals (Shanghai, China). RAGE antibody 553030 was purchased from Calbiochem (Merck, Darmstadt, Germany). Anti-HMGB1 polyclonal antibody 326052233 was purchased from SHINO-TEST Corporation (Sagamihara City, Japan) and GAPDH was purchased from Proteintech, Wuhan, China. Gelatin was purchased from Amresco (Solon, OH, USA). Zymogram renaturing buffer and developing buffer were purchased from Novex (Carlsbad, CA, USA). FBS was purchased from Gibco (Grand Island, NY, USA), and other cell culture media and supplements were purchased from HyClone (Logan, UT, USA). All other chemicals were purchased from Sigma Chemical Co. (St. Louis, MO, USA).

### 2.3. Cisplatin and Procyanidin Administration

For testing the role of MM-9/2 in CICI, the animals were divided into two different groups, the vehicle group (Veh) and the cisplatin group (Cis). The total cumulative dose of cisplatin for each mouse was 34.5 mg/kg. Cisplatin (2.3 mg/kg, dissolved in saline) or vehicle (saline) was intraperitoneally (i.p.) injected for three cycles consisting of 5 daily injections followed by 5 days of rest, according to a previous study [25].

To test the therapeutic role of procyanidin in CICI, the animals were divided into 6 groups: the vehicle group (Veh), the cisplatin group (Cis), the cisplatin plus 10 procyanidin group (10 mg/kg, p.o.), the cisplatin plus 20 procyanidin group (20 mg/kg, p.o.), the cisplatin plus 40 procyanidin group (40 mg/kg, p.o.), and the 40 procyanidin group (40 mg/kg, p.o.). Procyanidin (10, 20, 40 mg/kg, p.o.) or vehicle (saline, p.o.) was administered 1 h before cisplatin treatment.

### 2.4. Fear Conditioning

Contextual fear conditioning was performed in an automated system (Med Associates, Inc., St. Albans, VT, USA) and consisted of a single exposure to a context (3 min) and a foot shock (2 s; 0.7 mA; constant current) [26]. After the fear conditioning paradigm, mice were scored for 3 min by returning to the same chamber. Context-dependent freezing was measured 24 h after training every 10th second over 180 s by an observer unaware of the experimental conditions and expressed as a percentage of the total number of observations.

### 2.5. The Object Recognition Test (ORT)

ORT was performed in an apparatus consisting of a circular (30 cm) white Plexiglas arena with white Plexiglas walls (40 cm high) and a black-plastic-covered floor. Each animal was placed in the arena without objects for habituation. The training session (familiar object) was carried out one hour later. Each mouse was placed in the arena with a pair of identical objects (A1 and A2). The animals were allowed to explore freely for 10 min in the training phase. The animal went to the testing session (5 min). Mice were placed in the arena with one object (A1 or A2, familiar object) and a new object (B, the novel object). The “discrimination index can quantify the preference of experimental animals for novel objects”. The “discrimination index” is generally expressed by D2 (d2), which is the exploration time of the animals for the novel objects during the test period and the exploration of familiar objects. Calculated by the number of times, the specific formula is: D2 = (N − F)/(N + F), where “N” indicates the number of times the animal explored the novel object during the test period and “F” means that the animal is familiar with the test period. The number of object explorations, the “discrimination index”, considers the different levels of exploration activity between animals. An ORT index is related to the level of working memory [27].

### 2.6. Gelatin Zymography

Animals were anesthetized, and the tissue was rapidly dissected and homogenized in 1% NP40 lysis as described previously [22]. Next, 300–500 μg of protein per lane was loaded into the wells of precast gels (8% polyacrylamide gels containing 0.1% gelatin). After electrophoresis, each gel was incubated with 50 mL of developing buffer for 48 h (37.5 °C) in a shaking bath. Then the gels were stained with Coomassie brilliant blue (1%, with 10% acetic acid, 10% isopropyl alcohol, diluted with dd H_2_O).

### 2.7. An In Vivo Evans Blue Assay to Test Brain–Blood Vessel Permeability

Evans Blue is a dye that binds albumin [28]. When the vascular permeability endothelial cells become permeable, albumin marked by Evans Blue can be detected. A 2% solution of Evans Blue in normal saline (4 mL/kg of body weight) was injected intraperitoneally for 1 h before the animals were sacrificed. Afterward, the mice were sacrificed, and the brain tissue was collected for further tests. The samples were then homogenized in PBS and centrifuged (15 min, 15,000 rcf, 4 °C). To each 500 μL aliquot, an equal amount of 50% trichloroacetic acid was added to the supernatant and then incubated (overnight, 4 °C). The samples were finally centrifuged (30 min, 15,000 rcf, 4 °C) and were measured by spectrophotometer (RF-540, Shimadzu Corporation, Tokyo, Japan) at 632 nm. The results are presented as Evans Blue stain)/(g of tissue).

### 2.8. Cell Preparation and Stimulation

SHSY5Y cells and RAW264.7 cells were maintained in humidified 5% CO_2_ at 37 °C in Dulbecco’s modified Eagle’s Medium (DMEM) supplemented with 10% (*v*/*v*) FBS, penicillin (100 U/mL), and streptomycin (100 U/mL). For inducing inflammasome activation, cells were plated in a 6-well plate overnight. The medium was changed to serum-free medium, and then the SHSY5Y cells were treated with cisplatin (1 µM) for 8 h [29]. Then, we changed Dulbecco’s modified Eagle’s Medium (DMEM) supplemented with 10% (*v*/*v*) FBS and continued to culture for 8 h. The cell supernatants of SHSY5Y cells were collected at 0 h, 0.5 h, 1 h, 2 h, 4 h, and 8 h for further HMGB 1 and GAPDH Western blot testing. After 16 h, we added cell supernatant of SHSY5Y cells (the cells which have been activated and release a high level of HMGB-1 in a time-dependent manner) to RAW264.7 cells. TRL-4 inhibitor 10 μM, PI3K inhibitor 10 μM, Akt inhibitor 1 μM, and RAGE inhibitor 10 μM were used in the experiments. Then RAW264.7 cell supernatants were analyzed by gelatin zymography.

### 2.9. Western Blot

The cell supernatants of SHSY5Y cells were collected and homogenized in RIPA Lysis. The protein concentrations were determined by BCA Protein Assay (Thermo Fisher, Waltham, MA, USA). SDS-PAGE was used for separating different weight proteins by loading 30–60 μg of proteins in each gel. Then the separated proteins were electrophoretically transferred onto polyvinylidene fluoride membranes (Millipore Corp., Bedford, MA, USA). The membranes containing the target proteins were blocked with 5% bovine serum albumin for 2 h at room temperature, probed with antibodies overnight at 4 °C with the primary antibodies, and then incubated with HRP-coupled secondary antibodies. The primary antibodies used anti-HMGB1 (1:1000) and GAPDH (1:2000). Data were acquired with the Molecular Imager (Gel DocTM XR, 170-8170) and analyzed with Quantity One-4.6.5 (Bio-Rad Laboratories, Berkeley, CA, USA).

### 2.10. Data Analysis

Prism 9.0 for Mac (GraphPad, La Jolla, CA, USA) was used to calculate the mean and SEM and perform statistical analysis. We analyzed multiple groups means by one-way or two-way ANOVA, followed by Tukey’s multiple comparisons test wherever appropriate. We used the unpaired *t*-test for a two-group means comparison. *p* values less than 0.05 were considered significant.

## 3. Results

### 3.1. Repeated Treatment of Cisplatin-Induced Cognitive Impairment, Blood–Brain-Barrier Impairment, and Upregulation of MMP-9 Activity in the Hippocampus

**Figure 1:**
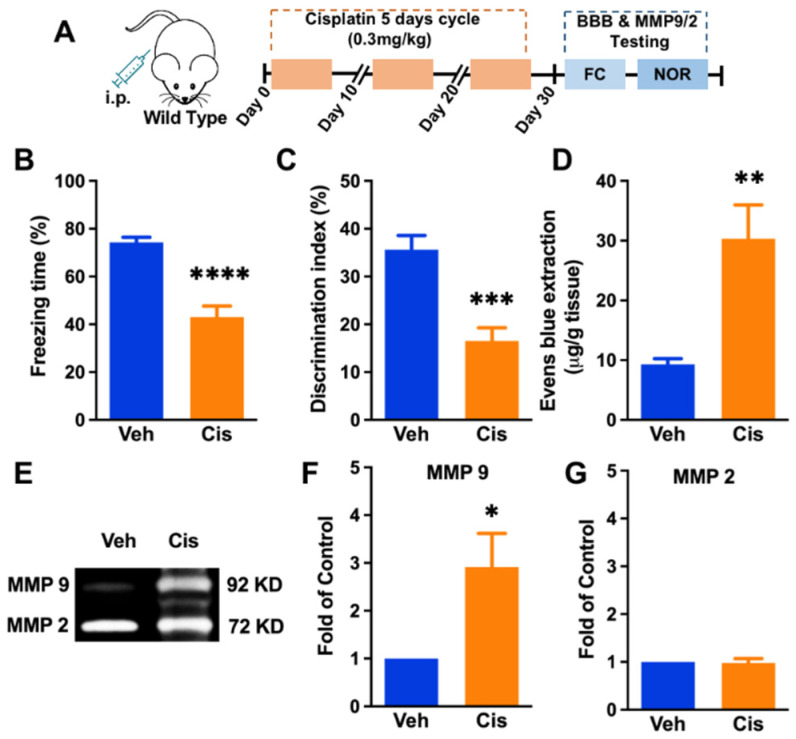
Repeated treatment of cisplatin-induced cognitive impairment, BBB interruption, and increased level of hippocampal MMP-9. (**A**) Behavior paradigm. (**B**) The deficiency in freezing behavior following cisplatin repeated infusion. *n* = 8. (**C**) The deficiency in the novel object recognition test following cisplatin repeated infusion. *n* = 8. (**D**) The Evans Blue extraction value in hippocampus tissue. *n* = 5. (**E**) Gelatin zymography shows activity changes of hippocampal MMP-9 and MMP-2 induced by cisplatin. (**F**) The fold of MMP-9 changes. (**G**) The fold of MMP-2 changes. *n* = 5, unpaired *t*-test (*p* * < 0.05, *p* ** < 0.01, *p* *** < 0.001, *p* **** < 0.0001 cisplatin vs. vehicle).

To confirm whether repeated usage of cisplatin can impair cognitive function, we conducted two different behavioral assessments in mice with or without cisplatin. We observed a significant memory impairment in the cisplatin group (unpaired *t*-test, *p* < 0.0001, Figure 1B) compared to the vehicle group by conducting a fear conditioning behavior test. We further conducted ORT for measuring hippocampal-dependent short-term working memory. In line with the fear conditioning test results, cisplatin also impaired short-term working memory (unpaired *t*-test, *p* = 0.0003, Figure 1C). We further evaluate the BBB function via examining Evans Blue leakage in the hippocampus. The results revealed that cisplatin results in an increased level of Evans Blue extraction (unpaired *t*-test, *p* = 0.0065, Figure 1D). We were interested in why the cisplatin-induced destruction of the BBB occurred. MMP9/2 was suggested to disrupt the BBB function in many diseases. Therefore, we explored the changes of MMP-9/2 in the hippocampus. Interestingly, cisplatin also increased MMP-9 activation significantly (*p* = 0.0262 Figure 1E,F), while there was no change in MMP-2 activation between groups (Figure 1G).

**Figure 2:**
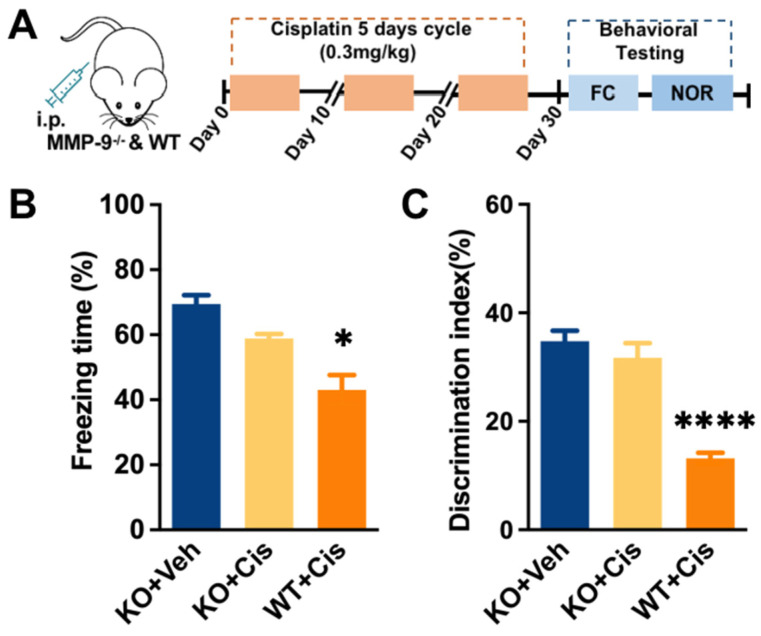
Effects of targeted system mutation of MMP-9 on cisplatin-induced memory impairment. (**A**) Behavior paradigm. (**B**) Effects of targeted mutation of MMP-9 on cisplatin-induced deficiency in freezing behavior. *n* = 5~8. (**C**) Effects of targeted mutation of MMP-9 on cisplatin-induced deficiency in the object recognition test. *n* = 5~8. Significance difference was revealed following one-way ANOVA (*p* * < 0.05, *p* **** < 0.0001 KO + cisplatin vs. KO + vehicle, Tukey post hoc tests).

These data revealed that MMP-9, not MMP-2, may contribute to the cisplatin-induced BBB damage. We further involved targeted systematic mutation of MMP-9 mice to confirm the hypothesis. Compared to that of the WT mice, repeated cisplatin treatment only induced a slight context-related memory loss in MMP-9^−/−^ mice (one-way ANOVA, *p* = 0.0236, Figure 2B). In line with the fear conditioning test results, cisplatin also impaired short-term working memory (one-way ANOVA, *p* < 0.0001, Figure 2C). These results further confirm the critical role of MMP-9 in cisplatin-related cognitive impairment.

### 3.2. Peripheral Inhibition of HMGB1 Significantly Prevents MMP-9 Increase

**Figure 3:**
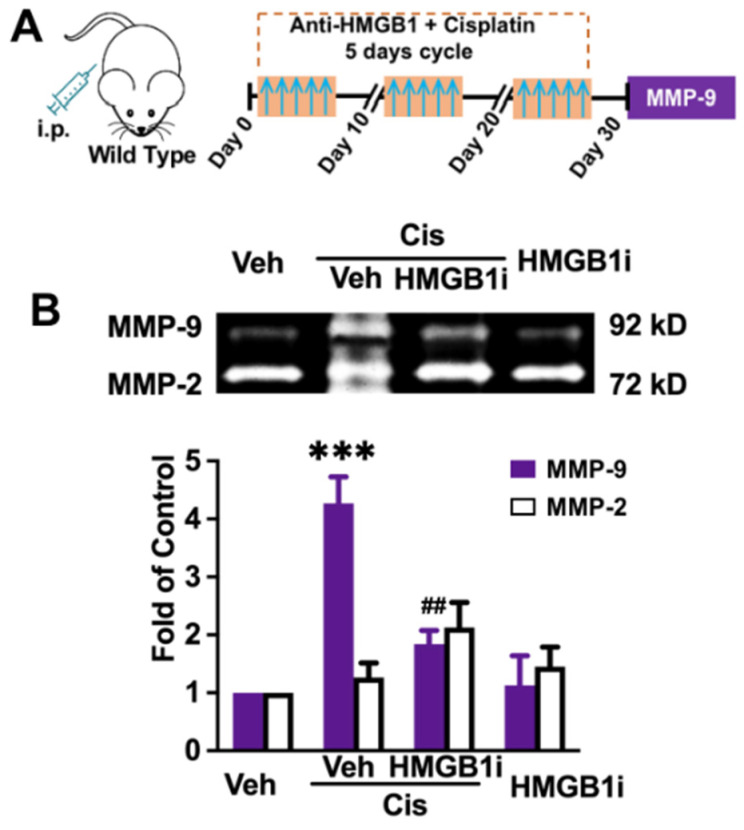
Effects of systemic HMGB1 neutralization on the cisplatin-induced increase of MMP-9 in the hippocampus. (**A**) Behavior paradigm. (**B**) The effect of anti-HMGB1 on cisplatin-induced increase of MMP-9 in the hippocampus. Up, the sample graph of Gelatin zymography, down, the analysis data. Significant difference was revealed following one-way ANOVA (*p* *** < 0.001 vs. vehicle, *p* ^##^ < 0.01, vs. cisplatin + vehicle group. Turkey post hoc tests). *n* = 3~4.

The reasons for MMP-9 upregulation after cisplatin repeated administration are what we were concerned about. As one of the cytotoxic agents, cisplatin can induce cell death and HMGB1 often increases rapidly [30]. Studies demonstrated it could induce MMP-9 release. We thus examined the role of systemic HMGB1 neutralization in preventing the increase of cisplatin-related MMP-9. The data show that cisplatin induced the increased level of MMP-9 (*p* = 0.0003, Figure 3B) and the preinhibition of HMGB1 significantly suppressed the level of MMP-9 in the hippocampus (*p* = 0.0056, Figure 3B). It suggests that circulating HMGB1 is the source of MMP-9.

### 3.3. The HMGB1/TLR4/PI3K/Akt Axis Is Involved in Activating MMP-9 in Raw 264.7 Cells In Vitro

**Figure 4:**
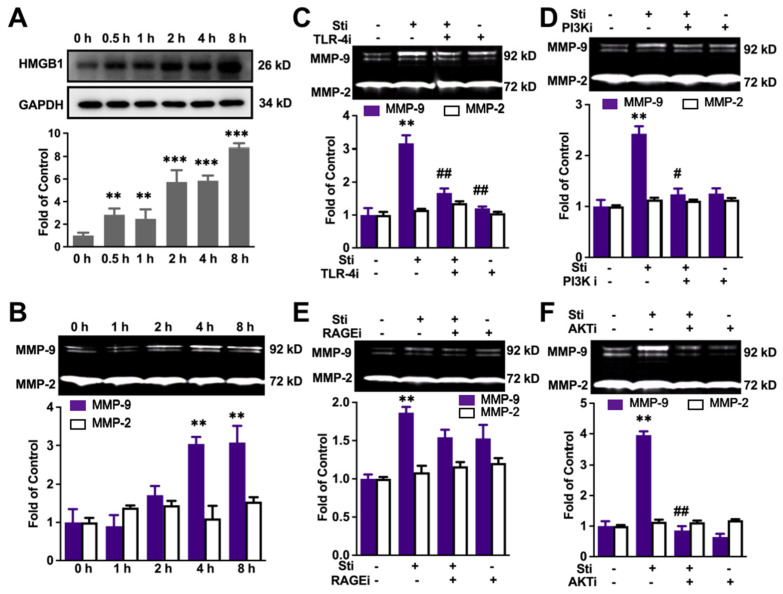
The increase of MMP-9 in cultured macrophages depends on the HMGB-1/TLR-4/PI3K/Akt pathway. (**A**) Cisplatin-induced increase of HMGB-1 in a time-dependent manner in cultured SH-SY5Y cells. (**B**) Stimulated supernatant induced an increase of MMP-9, but not MMP2, in a time-dependent manner in cultured Raw 264.7 cells. (**C**) TLR-4 inhibitor suppressed MMP-9 increase in Raw 264.7 cells. (**D**) The role of RAGE inhibitor in MMP-9 in Raw 264.7 cells. (**E**) IP3K inhibitor suppressed MMP-9 increase in Raw 264.7 cells. (**F**) Akt inhibitor suppressed MMP-9 increase in Raw 264.7 cells. The significant difference was revealed following one-way or two-way ANOVA (*p* ** < 0.05, *p* *** < 0.001 vs. control or 0 h. *p* ^#^ < 0.05, *p* ^##^ < 0.01 vs. stimuli group. Tukey post hoc tests). (**A**) *n* = 5; (**B**–**F**) *n* = 4.

MMP-9 could be released by many kinds of cells, while bone-marrow-derived macrophages (BM-DM) contain TLR-4 and are activated by HMGB 1 [31,32]. We thus explore the cell signaling mechanisms related to HMGB1-mediated MMP-9 in macrophages. To highly mimic the activation of macrophages in vivo, we used supernatant of the cisplatin-activated SH-SY5Y cells containing high levels of HMGB1 to stimulate Raw 264.7 cells. Firstly, we measured HMGB-1 in the supernatant of SH-SY5Y cells. The story of HMGB1 increased in a time-dependent manner in SH-SY5Y cells (F (5, 18) = 77.78, *p* < 0.0001) (Figure 4A). Next, to explore the mechanism of HMGB1-mediated MMP-9 activation from Raw 264.7 cells, as shown in Figure 4B, stimulated supernatant induced the release of MMP-9 in a time-dependent manner, as well (F (4, 40) = 41.59, *p* < 0.0001). TLR4 inhibitor TAK-242 (10 μM) significantly abolished the increase of MMP-9, and a similar suppression roles of the PI3K inhibitor AS-605240 (10 μM) and the Akt inhibitor GSK690693 (1 μM) were observed. However, RAGE inhibitor 553030 (10 μM) did not block that increase (Figure 4C–F).

These data suggest MMP-9 secretion in macrophages via the HMGB1/TLR4/PI3K/Akt pathway.

### 3.4. Procyanidin Suppressed MMP-9 Activity, the BBB Interruption, and Cisplatin-Induced Cognitive Decline in a Dose-Dependent Manner

**Figure 5:**
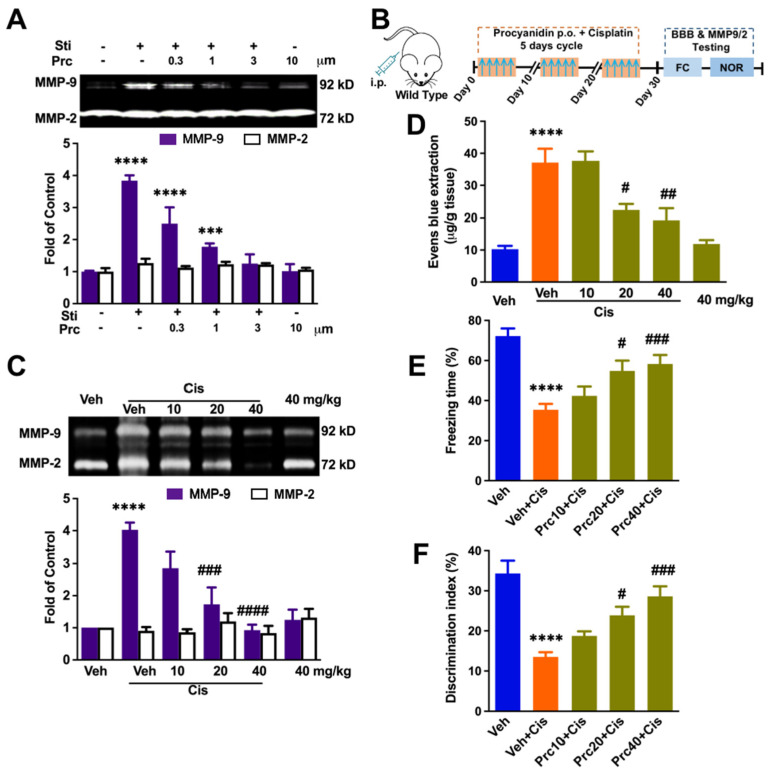
Prophylactic procyanidin dose-dependent rescue of cisplatin-induced increased levels of MMP-9, BBB damage, and cognitive decline. (**A**) Procyanidin suppressed the release of MMP-9 of Raw 264.7 cells induced by the supernatant containing high levels of HMGB1. *n* = 4. (**B**) Behavior paradigm. (**C**) Procyanidin suppressed the cisplatin-induced increase of MMP-9 in the hippocampus in a dose-dependent manner. *n* = 4~5. (**D**) Evans Blue extraction showing the effects of procyanidin on the BBB permeability changes. *n* = 4. (**E**) Procyanidin rescues cisplatin-induced deficiency of freezing score in a dose-dependent manner. *n* = 8. (**F**) Procyanidin rescues cisplatin-induced deficiency of new object recognition in a dose-dependent manner. *n* = 8. Difference was revealed following one-way or two-way ANOVA (*p* **** < 0.0001, *p* *** < 0.001 vs. control. *p* ^#^ < 0.05, *p* ^##^ < 0.01, *p* ^###^ < 0.001, *p* ^####^ < 0.0001 vs. cisplatin group. Tukey post hoc tests).

It was found that procyanidin can suppress MMP-9 and is a safe and effective therapeutic method for neuropathic pain and CIPN [14,22]. We therefore determined its effect on MMP-9 activity in vivo. As shown in Figure 5A, procyanidin could markedly decrease the activity of MMP-9 in cultured Raw 264.7 cells (F (5, 36) = 55.20, *p* < 0.0001). Further, the effects of procyanidin on MMP-9/2 were confirmed in vitro. Procyanidin (10, 20, 40 mg/kg, p.o.) was given one hour before each cisplatin injection. The data demonstrated that procyanidin (20, 40 mg/kg, p.o.) suppressed cisplatin-related MMP-9 activation (F (5, 22) = 17.73, *p* < 0.0001, Figure 5C). Further, we investigate the effects of procyanidin on BBB interruptions and its therapeutic roles in CICI. The data showed that oral procyanidin attenuated the CICI in a dose-dependent manner (F (5, 18) = 18.52, *p* < 0.0001, Figure 5D).

Finally, we evaluated the role of procyanidin on the repeated-cisplatin-administration-induced memory decline. Fear conditioning tests and ORT were performed. The results showed that prophylactic procyanidin administration could rescue the deficiency of freezing scores in a dose-dependent manner (F (4, 35) = 11.34, *p* < 0.0001, Figure 5E). In addition, a similar dose-dependent inhibition role of procyanidin was observed by ORT data (F (4, 34) = 14.27, *p* < 0.0001, Figure 5F).

Therefore, prophylactic procyanidin administration rescues the cognitive impairment caused by cisplatin, which may be achieved by inhibiting MMP-9 expression and BBB damage.

## 4. Discussion

This study found that grape-seed-orientated procyanidin significantly suppressed MMP-9 activation. Prophylactic procyanidin administration attenuated the cognitive impairment induced by repeated cisplatin injection in a dose-dependent manner.

A growing number of long-term cancer survivors has brought awareness to the side effects of chemotherapy. It has been reported that the administration of cisplatin could induce acute kidney injury, cognitive impairment, hearing loss, and peripheral neuropathy [33,34]. There is an urgent need to explore new, safe, and clinical drugs to prevent the CICI [20].

Traditionally, the chemotherapy agents usually cannot cross the BBB and, therefore, many physicians ignore the cognitive declines. However, new evidence showed that systemic chemotherapy drugs are neurotoxic and lead to cognitive decline [35]. Some brain regions, such as the hippocampus, amygdala, lateral ventricles, and superior temporal regions, are related to mild cognitive impairment (MCI) and Alzheimer’s disease (AD) [36]. The hippocampus is one of the most vulnerable brain regions linked to cognitive impairment. Interestingly, it showed that cisplatin could penetrate the BBB and persist a long time in the hippocampus, as well. These data raised our interest, and we then tested the permeability of the BBB in the hippocampus after cisplatin administration. Clinical and research studies revealed the importance of hippocampal neurotransmitter systems in cognition alteration [37,38]. Moreover, cisplatin interrupts the interaction between the excitatory and inhibitory neurotransmitter systems during the postnatal maturation of cells [39]. Other pentameric neurotransmitters or neurotrophic factors, such as the brain-derived neurotrophic factor (BDNF), could also be suppressed by the cisplatin [40]. Thus, the neurotransmitter systems might be the potential targets of cisplatin.

As the most critical molecule, MMP-9/2 damages BBB integrity and involves several pathological processes of cognitive impairment diseases. It also increased dramatically in dorsal root ganglia in CIPN. We found that cisplatin significantly increased the level of MMP-9 in the hippocampus, and systemic knockout of MMP-9 could rescue cisplatin-related working memory decline.

We are interested in the source of MMP-9. MMP-9 is secreted by many cell types, including neutrophils, macrophages, and fibroblasts. As a DNA targeting agent, systemic cisplatin administration induces DNA damage and apoptosis of tumor cells and other cells. The immune cells could be activated by the DAMPs, such as HMGB1, released from apoptosis and other damaged cells [41]. Of all types of cells, HMGB1 can be released into the extracellular environment and shuttle freely between the nucleus and cytoplasm [42]. Thus, it is believed that HMGB1 induces the MMP-9 upstream.

Our data showed that pretreated mice with the HMGB1 neutralizing antibody could remarkably decrease the level of hippocampal MMP-9. Cisplatin could induce SH-SY5Y cells to release HMGB1 in a time-dependent manner. The stimulated SH-SY5Y cells supernatant successfully induced the macrophage Raw 264.7 cell releasing MMP-9. HMGB1 can bind to cell surface receptors, including the receptor for advanced glycation end products (RAGE) and TLR2 and TLR4 [43]. By binding to cellular receptors, including Toll-like receptors (TLR)-2 and TLR-4, HMGB1 controls inflammatory reactions and MMP-9 releasing. RAGE is the natural receptor for HMGB1, and its activation leads to intracellular signaling pathway activation, such as nuclear factor-kappa B (NF-κB) and PI3K/Akt [44,45]. The PI3K/Akt signaling pathway is involved in many biological processes, including cell growth and apoptosis. Recently, the PI3K/Akt signaling pathway has been critical in chemokine-induced EPC migration [46]. Thus, we used the TLR-4 inhibitor, PI3K inhibitor, Akt inhibitor, and RAGE antibody to confirm the role of HMGB1 in MMP-9 upregulation. The data indicated that TLR-4, but not RAGE, is downstream of HMGB1-induced MMP-9 releasing, and PI3K/Akt plays a crucial role.

Previous studies have found that the grape seed extract procyanidin is a safe and effective therapeutic method for many kinds of diseases. It could protect granulosa cells from oxidative injury and suppress neuropathic pain and CIPN by inhibiting MMP-9 [14,22,47]. It also inhibits the in vitro growth and invasion of pancreatic carcinoma cells by suppressing MMP-9 and MMP-2 [48]. We therefore determined its effect on MMP-9 activity in vivo. Prophylactic procyanidin could markedly decrease the activity of MMP-9 in cultured Raw 264.7 cells. Procyanidin (20, 40 mg/kg, p.o.) suppressed cisplatin-related MMP-9 level increase, BBB interruptions, and cognitive impairment caused by cisplatin. The oral LD50 values of procyanidin are over 4000 mg/kg in mice. In our study, the dose applied in our experiment (the highest, 40 mg/kg, p.o.) is reasonably believed to be safe. Our results indicated that procyanidin’s grape seed extract may protect BBB function by inhibiting MMP-9.

We believe that the grape seed neuroprotective action that finally resulted in BBB function protection and attenuated CICI is mainly through the inhibition of MMP-9. Immune dysregulation is possibly one of the proposed pathogeneses of CICI, with the release of inflammatory mediators in response to chemotherapeutic drugs or tumors. As a result of chemotherapy, the periphery releases inflammatory cytokines that cross the blood–brain barrier, including TNF-α, interleukin-1, and IL-6 [49]. It is well-known that MMP-9 could cleave vascular basal lamina and tight junctions between cells and enhance BBB permeability [11,12]. MMP-9 plays a prominent role in the transmigration of immune cells (T cells and macrophages) to various tissues, including the brain [13]. In addition, the MMP-9 produced by macrophages promotes inflammatory cell infiltration, and its role in neuroinflammation has been well-reviewed [12]. Thus, the procyanidin given prior to cisplatin might suppress MMP-9 release from systematic macrophages and result in inflammatory cytokine release. Future studies should evaluate the action of the molecular mechanism of procyanidin in MMP-9 inhibition.

Further, several natural products with neuroprotective properties were confirmed to ameliorate cisplatin neurotoxic action [7]. N-acetylcysteine prevented free radical production and ameliorated apoptotic cell death associated with cisplatin [19]. Ginsenoside Rg1 could prevent CICI by inhibiting microglia-mediated cytokine release and neuroplasticity [20]. Curcumin, a natural product derived from the root of the plant *Curcuma longa*, attenuates cognitive impairment by enhancing autophagy in chemotherapy [21]. While the present study suggested the effectiveness of preadministrated procyanidin in attenuating CICI, the proper dose and other related side effects of procyanidin should be carefully evaluated when generalizing to clinical settings. For example, the precise dose of it needs to be evaluated among patients. Thus, clinical studies could focus on the impact of the combined usage of natural products (extract segments) with chemotherapeutic drugs in alleviating cisplatin-induced neurotoxicity. Although we believe that prophylactic usage of procyanidin may achieve better effect, we have not tested the design of prophylactic groups for further study. Future research should compare the effects of prophylactic procyanidin and the coadministration of it on counteracting chemotherapy-induced changes to BBB permeability and cognitive decline.

## 5. Conclusions

In the study, we found that cisplatin could induce the upregulation of MMP-9 and BBB interruption. Procyanidin can suppress the increased level of MMP-9 and BBB interruption. The HMGB1/PI3K/Akt signaling pathway may contribute to the upregulation of MMP-9 in macrophages. Procyanidin could be a potential supplemental drug for the management of chemotherapy.

## Data Availability

The data presented in this study are available on request from the corresponding authors.

## References

[1] Dias-Carvalho A., Ferreira M., Ferreira R., Bastos M.L., Sa S.I., Capela J.P., Carvalho F., Costa V.M. (2022). Four decades of chemotherapy-induced cognitive dysfunction: Comprehensive review of clinical, animal and in vitro studies, and insights of key initiating events. Arch. Toxicol..

[2] Vichaya E.G., Chiu G.S., Krukowski K., Lacourt T.E., Kavelaars A., Dantzer R., Heijnen C.J., Walker A.K. (2015). Mechanisms of chemotherapy-induced behavioral toxicities. Front. Neurosci..

[3] Simo M., Rifa-Ros X., Rodriguez-Fornells A., Bruna J. (2013). Chemobrain: A systematic review of structural and functional neuroimaging studies. Neurosci. Biobehav. Rev..

[4] Ahles T.A., Saykin A.J., McDonald B.C., Li Y., Furstenberg C.T., Hanscom B.S., Mulrooney T.J., Schwartz G.N., Kaufman P.A. (2010). Longitudinal assessment of cognitive changes associated with adjuvant treatment for breast cancer: Impact of age and cognitive reserve. J. Clin. Oncol..

[5] Fuertes M.A., Castilla J., Alonso C., Perez J.M. (2003). Cisplatin biochemical mechanism of action: From cytotoxicity to induction of cell death through interconnections between apoptotic and necrotic pathways. Curr. Med. Chem..

[6] George R.P., Semendric I., Hutchinson M.R., Whittaker A.L. (2021). Neuroimmune reactivity marker expression in rodent models of chemotherapy-induced cognitive impairment: A systematic scoping review. Brain. Behav. Immun..

[7] Ongnok B., Chattipakorn N., Chattipakorn S.C. (2020). Doxorubicin and cisplatin induced cognitive impairment: The possible mechanisms and interventions. Exp. Neurol..

[8] Oppegaard K., Harris C.S., Shin J., Paul S.M., Cooper B.A., Chan A., Anguera J.A., Levine J., Conley Y., Hammer M. (2021). Cancer-related cognitive impairment is associated with perturbations in inflammatory pathways. Cytokine.

[9] Simic G., Spanic E., Langer Horvat L., Hof P.R. (2019). Blood-brain barrier and innate immunity in the pathogenesis of Alzheimer’s disease. Prog. Mol. Biol. Transl. Sci..

[10] Sweeney M.D., Zhao Z., Montagne A., Nelson A.R., Zlokovic B.V. (2019). Blood-Brain Barrier: From Physiology to Disease and Back. Physiol. Rev..

[11] Vafadari B., Salamian A., Kaczmarek L. (2016). MMP-9 in translation: From molecule to brain physiology, pathology, and therapy. J. Neurochem..

[12] Vandooren J., Van Damme J., Opdenakker G. (2014). On the structure and functions of gelatinase B/matrix metalloproteinase-9 in neuroinflammation. Prog. Brain Res..

[13] Yong V.W., Krekoski C.A., Forsyth P.A., Bell R., Edwards D.R. (1998). Matrix metalloproteinases and diseases of the CNS. Trends Neurosci..

[14] Gu H., Wang C., Li J., Yang Y., Sun W., Jiang C., Li Y., Ni M., Liu W.T., Cheng Z. (2020). High mobility group box-1-toll-like receptor 4-phosphatidylinositol 3-kinase/protein kinase B-mediated generation of matrix metalloproteinase-9 in the dorsal root ganglion promotes chemotherapy-induced peripheral neuropathy. Int. J. Cancer.

[15] Fumagalli G., Monza L., Cavaletti G., Rigolio R., Meregalli C. (2020). Neuroinflammatory Process Involved in Different Preclinical Models of Chemotherapy-Induced Peripheral Neuropathy. Front. Immunol..

[16] Zhang Q., Raoof M., Chen Y., Sumi Y., Sursal T., Junger W., Brohi K., Itagaki K., Hauser C.J. (2010). Circulating mitochondrial DAMPs cause inflammatory responses to injury. Nature.

[17] Zhu X., Cong J., Yang B., Sun Y. (2020). Association analysis of high-mobility group box-1 protein 1 (HMGB1)/toll-like receptor (TLR) 4 with nasal interleukins in allergic rhinitis patients. Cytokine.

[18] Yang H., Hreggvidsdottir H.S., Palmblad K., Wang H., Ochani M., Li J., Lu B., Chavan S., Rosas-Ballina M., Al-Abed Y. (2010). A critical cysteine is required for HMGB1 binding to Toll-like receptor 4 and activation of macrophage cytokine release. Proc. Natl. Acad. Sci. USA.

[19] Lomeli N., Di K., Czerniawski J., Guzowski J.F., Bota D.A. (2017). Cisplatin-induced mitochondrial dysfunction is associated with impaired cognitive function in rats. Free Radic. Biol. Med..

[20] Shi D.D., Huang Y.H., Lai C.S.W., Dong C.M., Ho L.C., Li X.Y., Wu E.X., Li Q., Wang X.M., Chen Y.J. (2019). Ginsenoside Rg1 Prevents Chemotherapy-Induced Cognitive Impairment: Associations with Microglia-Mediated Cytokines, Neuroinflammation, and Neuroplasticity. Mol. Neurobiol..

[21] Yi L.T., Dong S.Q., Wang S.S., Chen M., Li C.F., Geng D., Zhu J.X., Liu Q., Cheng J. (2020). Curcumin attenuates cognitive impairment by enhancing autophagy in chemotherapy. Neurobiol. Dis..

[22] Li J., Xu L., Deng X., Jiang C., Pan C., Chen L., Han Y., Dai W., Hu L., Zhang G. (2016). N-acetyl-cysteine attenuates neuropathic pain by suppressing matrix metalloproteinases. Pain.

[23] Chen L., You Q., Hu L., Gao J., Meng Q., Liu W., Wu X., Xu Q. (2017). The Antioxidant Procyanidin Reduces Reactive Oxygen Species Signaling in Macrophages and Ameliorates Experimental Colitis in Mice. Front. Immunol..

[24] D’Eliseo D., Pannucci E., Bernini R., Campo M., Romani A., Santi L., Velotti F. (2019). In vitro studies on anti-inflammatory activities of kiwifruit peel extract in human THP-1 monocytes. J. Ethnopharmacol..

[25] Zhou W., Kavelaars A., Heijnen C.J. (2016). Metformin Prevents Cisplatin-Induced Cognitive Impairment and Brain Damage in Mice. PLoS ONE.

[26] Gao C., Frausto S.F., Guedea A.L., Tronson N.C., Jovasevic V., Leaderbrand K., Corcoran K.A., Guzman Y.F., Swanson G.T., Radulovic J. (2011). IQGAP1 regulates NR2A signaling, spine density, and cognitive processes. J. Neurosci..

[27] Ahuja M., Buabeid M., Abdel-Rahman E., Majrashi M., Parameshwaran K., Amin R., Ramesh S., Thiruchelvan K., Pondugula S., Suppiramaniam V. (2017). Immunological alteration & toxic molecular inductions leading to cognitive impairment & neurotoxicity in transgenic mouse model of Alzheimer’s disease. Life Sci..

[28] Manaenko A., Chen H., Kammer J., Zhang J.H., Tang J. (2011). Comparison Evans Blue injection routes: Intravenous versus intraperitoneal, for measurement of blood-brain barrier in a mice hemorrhage model. J. Neurosci. Methods.

[29] Andres A.L., Gong X., Di K., Bota D.A. (2014). Low-doses of cisplatin injure hippocampal synapses: A mechanism for ‘chemo’ brain?. Exp. Neurol..

[30] Chavez-Dominguez R.L., Perez-Medina M.A., Lopez-Gonzalez J.S., Galicia-Velasco M., Matias-Florentino M., Avila-Rios S., Rumbo-Nava U., Salgado-Aguayo A., Gonzalez-Gonzalez C., Aguilar-Cazares D. (2021). Role of HMGB1 in Cisplatin-Persistent Lung Adenocarcinoma Cell Lines. Front. Oncol..

[31] Degos V., Vacas S., Han Z., van Rooijen N., Gressens P., Su H., Young W.L., Maze M. (2013). Depletion of bone marrow-derived macrophages perturbs the innate immune response to surgery and reduces postoperative memory dysfunction. Anesthesiology.

[32] Terrando N., Eriksson L.I., Ryu J.K., Yang T., Monaco C., Feldmann M., Jonsson Fagerlund M., Charo I.F., Akassoglou K., Maze M. (2011). Resolving postoperative neuroinflammation and cognitive decline. Ann. Neurol..

[33] Shi M., Maique J., Shepard S., Li P., Seli O., Moe O.W., Hu M.C. (2022). In vivo evidence for therapeutic applications of beclin 1 to promote recovery and inhibit fibrosis after acute kidney injury. Kidney Int..

[34] Ma J., Goodwani S., Acton P.J., Buggia-Prevot V., Kesler S.R., Jamal I., Mahant I.D., Liu Z., Mseeh F., Roth B.L. (2021). Inhibition of dual leucine zipper kinase prevents chemotherapy-induced peripheral neuropathy and cognitive impairments. Pain.

[35] Geraghty A.C., Gibson E.M., Ghanem R.A., Greene J.J., Ocampo A., Goldstein A.K., Ni L., Yang T., Marton R.M., Pasca S.P. (2019). Loss of Adaptive Myelination Contributes to Methotrexate Chemotherapy-Related Cognitive Impairment. Neuron.

[36] Morrison C., Dadar M., Shafiee N., Villeneuve S., Louis Collins D., for Alzheimer’s Disease Neuroimaging Initiative (2022). Regional brain atrophy and cognitive decline depend on definition of subjective cognitive decline. Neuroimage Clin..

[37] Pereira A.C., Lambert H.K., Grossman Y.S., Dumitriu D., Waldman R., Jannetty S.K., Calakos K., Janssen W.G., McEwen B.S., Morrison J.H. (2014). Glutamatergic regulation prevents hippocampal-dependent age-related cognitive decline through dendritic spine clustering. Proc. Natl. Acad. Sci. USA.

[38] Liu A.K.L., Chau T.W., Lim E.J., Ahmed I., Chang R.C., Kalaitzakis M.E., Graeber M.B., Gentleman S.M., Pearce R.K.B. (2019). Hippocampal CA2 Lewy pathology is associated with cholinergic degeneration in Parkinson’s disease with cognitive decline. Acta Neuropathol. Commun..

[39] Piccolini V.M., Cerri S., Romanelli E., Bernocchi G. (2012). Interactions of neurotransmitter systems during postnatal development of the rat hippocampal formation: Effects of cisplatin. Exp. Neurol..

[40] Sun Y.X., Yang J., Wang P.Y., Li Y.J., Xie S.Y., Sun R.P. (2013). Cisplatin regulates SH-SY5Y cell growth through downregulation of BDNF via miR-16. Oncol. Rep..

[41] Scaffidi P., Misteli T., Bianchi M.E. (2002). Release of chromatin protein HMGB1 by necrotic cells triggers inflammation. Nature.

[42] Gardella S., Andrei C., Ferrera D., Lotti L.V., Torrisi M.R., Bianchi M.E., Rubartelli A. (2002). The nuclear protein HMGB1 is secreted by monocytes via a non-classical, vesicle-mediated secretory pathway. EMBO Rep..

[43] Wang H., Yang H., Czura C.J., Sama A.E., Tracey K.J. (2001). HMGB1 as a late mediator of lethal systemic inflammation. Am. J. Respir. Crit. Care Med..

[44] Toure F., Zahm J.M., Garnotel R., Lambert E., Bonnet N., Schmidt A.M., Vitry F., Chanard J., Gillery P., Rieu P. (2008). Receptor for advanced glycation end-products (RAGE) modulates neutrophil adhesion and migration on glycoxidated extracellular matrix. Biochem. J..

[45] Kim J., Park J.C., Lee M.H., Yang C.E., Lee J.H., Lee W.J. (2017). High-Mobility Group Box 1 Mediates Fibroblast Activity via RAGE-MAPK and NF-kappaB Signaling in Keloid Scar Formation. Int. J. Mol. Sci..

[46] Zuccolo E., Di Buduo C., Lodola F., Orecchioni S., Scarpellino G., Kheder D.A., Poletto V., Guerra G., Bertolini F., Balduini A. (2018). Stromal Cell-Derived Factor-1alpha Promotes Endothelial Colony-Forming Cell Migration Through the Ca(2+)-Dependent Activation of the Extracellular Signal-Regulated Kinase 1/2 and Phosphoinositide 3-Kinase/AKT Pathways. Stem Cells Dev..

[47] Zhang J.Q., Wang X.W., Chen J.F., Ren Q.L., Wang J., Gao B.W., Shi Z.H., Zhang Z.J., Bai X.X., Xing B.S. (2019). Grape Seed Procyanidin B2 Protects Porcine Ovarian Granulosa Cells against Oxidative Stress-Induced Apoptosis by Upregulating let-7a Expression. Oxid. Med. Cell. Longev..

[48] Chung Y.C., Huang C.C., Chen C.H., Chiang H.C., Chen K.B., Chen Y.J., Liu C.L., Chuang L.T., Liu M., Hsu C.P. (2012). Grape-seed procyanidins inhibit the in vitro growth and invasion of pancreatic carcinoma cells. Pancreas.

[49] Mounier N.M., Abdel-Maged A.E., Wahdan S.A., Gad A.M., Azab S.S. (2020). Chemotherapy-induced cognitive impairment (CICI): An overview of etiology and pathogenesis. Life Sci..

